# Determination of critical cooling rates in metallic glass forming alloy libraries through laser spike annealing

**DOI:** 10.1038/s41598-017-07719-2

**Published:** 2017-08-02

**Authors:** Punnathat Bordeenithikasem, Jingbei Liu, Sebastian A. Kube, Yanglin Li, Tianxing Ma, B. Ellen Scanley, Christine C. Broadbridge, Joost J. Vlassak, Jonathan P. Singer, Jan Schroers

**Affiliations:** 1 Department of Mechanical Engineering and Materials Science, Yale University, New Haven, Connecticut 06511 USA; 2 Department of Mechanical and Aerospace Engineering, Rutgers University, Piscataway, New Jersey 08854 USA; 3 Department of Physics, Southern Connecticut State University, New Haven, Connecticut 06515 USA; 4 School of Engineering and Applied Science, Harvard University, Cambridge, Massachusetts 02138 USA

## Abstract

The glass forming ability (GFA) of metallic glasses (MGs) is quantified by the critical cooling rate (*R*_C_). Despite its key role in MG research, experimental challenges have limited measured *R*_C_ to a minute fraction of known glass formers. We present a combinatorial approach to directly measure *R*_C_ for large compositional ranges. This is realized through the use of compositionally-graded alloy libraries, which were photo-thermally heated by scanning laser spike annealing of an absorbing layer, then melted and cooled at various rates. Coupled with X-ray diffraction mapping, GFA is determined from direct *R*_C_ measurements. We exemplify this technique for the Au-Cu-Si system, where we identify Au_56_Cu_27_Si_17_ as the alloy with the highest GFA. In general, this method enables measurements of *R*_C_ over large compositional areas, which is powerful for materials discovery and, when correlating with chemistry and other properties, for a deeper understanding of MG formation.

## Introduction

The glass forming ability (GFA) is most directly quantified by the inverse of the critical cooling rate (*R*_C_), the minimum cooling rate required to avoid crystallization upon solidification resulting in vitrification into a fully amorphous state. Despite its significance, *R*_C_ has only been directly measured for a minute fraction of metallic glass (MG) forming systems^1–6^. The majority of *R*_C_ quantifications have been estimated using indirect methods. This is due to challenges in experimentally measuring *R*_C_, particularly for glass formers with *R*_C_ exceeding 10^4^ K s^−1^. Therefore, a method to directly measure *R*_C_ would be both scientifically and technologically powerful. Even more impactful would be the ability to measure *R*_C_ over large composition ranges as it would enhance alloy development and provide insight into the mechanistic origins of glass formation.

Strategies to effectively consider large numbers of alloys are based on combinatorial synthesis paired with high-throughput characterization of properties. Such combinatorial strategies, well established in pharmaceutical research^7^, are relatively new in materials science and particularly MG research. First combinatorial methods applied in MG research include measurements of mechanical properties^8–11^, thermophysical properties^12–14^, functional properties^15–17^, thermoplastic formability^18^, and microstructure evolution^11,12,19–21^. Attempts were made to draw conclusions about GFA from some of those measurements^10,12,18,21^. More recently approaches to measure GFA directly have been limited in cooling rate, in alloy composition, and generally in versatility^21–24^.

In this study, we use a combinatorial approach to directly measure *R*_C_ across a significant fraction of the composition space in a ternary system. Through indirect laser spike annealing (LSA), we melt and cool co-sputtered alloy libraries over a range of controlled cooling rates. Employing structural and chemical characterization, *R*_C_ as a function of composition is determined and Au_56_Cu_27_Si_17_ was identified as the alloy with the highest GFA in the Au-Cu-Si system.

## Results

### Experimental setup for *R*_C_ measurements using laser heating

**Figure 1:**
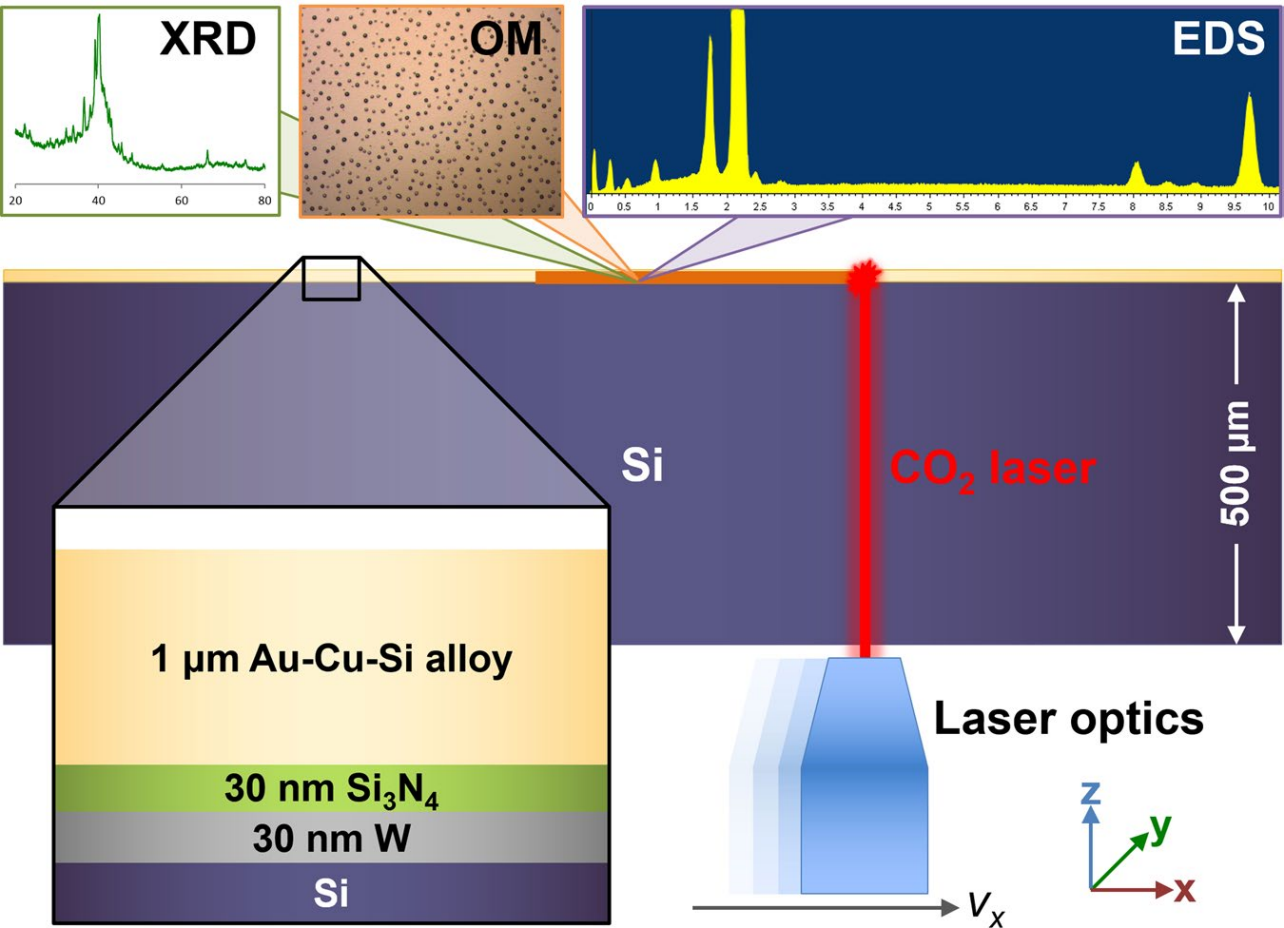
Schematic of the experimental setup for *R*_C_ measurements via laser spike annealing. We use a CO_2_ laser with a 60 W continuous wave (CW) maximum output, operating at a wavelength of 10.6 μm. Compatible with this wavelength, intrinsic (undoped) Si, which has low absorption at 10.6 μm^32,51^, is selected as the heat sink material. For the photo-thermal absorbing layer, we use 30 nm tungsten (W)^52^, which is electrically insulated with 30 nm Si_3_N_4_ to prevent reflective thick film behavior^53^ from the ~1 μm Au-Cu-Si ternary alloy library. This setup is mounted onto a fixture with adjustable height (*z*) for laser focus while the laser optics are motorized on two axes (*x* and *y*) such that the laser can be delivered to the substrate at known positions and scan speeds. Altering the laser scan speed, in this case *v*_x_, varies the temperature profile. The samples are characterized using XRD, OM, and EDS mapping.

To measure *R*_C_, the synthesized alloy library must be melted and cooled in a controlled manner. Using LSA as a heating method provides localized, concentrated heat flux with precise control over the heating and cooling profile^25–28^, which is ideal for combinatorial studies. However, directly exposing the laser onto an alloy film library can produce inconsistent temperature profiles. This is due to the composition-dependence of the absorption coefficient, which can vary significantly in a ternary system^29,30^. To avoid associated uncertainty in temperature and cooling rates, the alloy library is heated indirectly (Fig. 1). We realize this by co-sputtering the alloy library onto a substrate that is designed specifically for photo-thermal heating (see Methods). Such a substrate consists of a heat sink that is optically transparent to the laser and a photo-thermal absorbing layer. When the laser impinges on the substrate side, the radiation is transmitted through the heat sink and then absorbed by the absorbing layer, consequently heating the alloy via conduction. Tuning the incident laser heat flux via the laser scan speed determines the temperature profile, hence the cooling rate, experienced by the alloy.

### Determination of cooling rates

**Figure 2:**
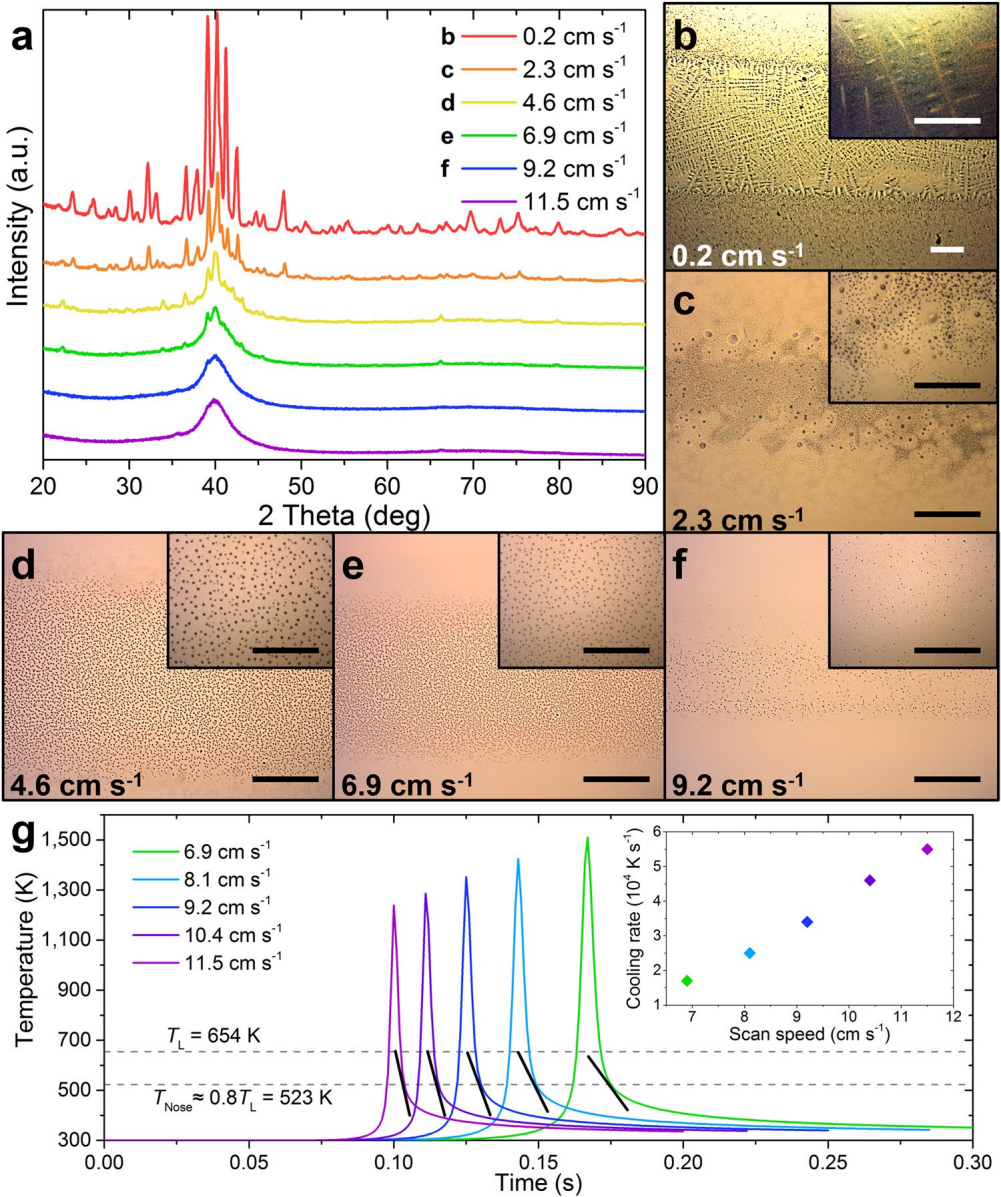
Determination of cooling rates at different laser scan speeds. (**a**) XRD diffractograms measured from Au_55_Cu_25_Si_20_ films melted using multiple laser scan speeds. With higher scan speeds, the amorphous fraction increases, indicating faster cooling rates with faster scan speeds. (**b–f**) OM images, with a high magnification inset, of the Au_55_Cu_25_Si_20_ film surface as a function of various laser scan speeds. Scale bars are 250 μm and 25 μm in the insets. For laser scan speeds exceeding 9.2 cm s^−1^, the features are not clearly resolved by OM. The heat affected zone size is measured from the extent over which the microstructural features are observed in the direction perpendicular to the laser scan. (**g**) Simulated temperature profiles of laser heating experiments at variable scan speeds. The simulation was fitted with experimental values of the measured heat affected zone size. The *T*_L_ was taken from measured values of bulk samples of Au_55_Cu_25_Si_20_^31^ and *T*_Nose_ was approximated to be 0.8*T*_L_. The inset shows the calculated cooling rate at 0.8*T*_L_ as a function of laser scan speed.

To demonstrate the proposed method, we first chose a film of constant composition Au_55_Cu_25_Si_20_ (Fig. 2)^31^. Its composition was verified by energy dispersive X-ray spectroscopy (EDS). To vary the temperature profiles, the laser translational scan speed (*v*_*x*_ in Fig. 1) was systematically changed. X-ray diffraction (XRD) measurements, utilizing a beam mask commensurate to the heat affected zone size (2 mm), and optical microscopy (OM) were then carried out *ex-situ* (Fig. 2). For the slowest scan speed of 0.2 cm s^−1^, large dendrites are observed (Fig. 2b) indicating melting and slow crystallization during cooling. XRD results reveal that with increasing laser scan speed, the amount of crystallinity and crystal size decreases.

To quantify the cooling rates for a given laser scan speed and power, simulations of the LSA process were conducted over the range of scan speeds where melting was visually observed and XRD measurements (Fig. 2a) on the remelted region indicated a transition from crystalline to amorphous. These simulations assumed a Gaussian-shaped heat source (see Methods). The laser power and peak width were determined by fitting the heat affected zone size, measured from OM images at different scan speeds (Fig. 2b–f), to produce the most representative simulations. The cooling rates could be extracted from the simulated temperature profiles of a particular point along the laser scan line (Fig. 2g). The cooling rate from the temperature profile that defines *R*_C_ is the cooling rate at the nose temperature (*T*_Nose_) of the time-temperature-transformation (TTT) diagram. Across multiple Au-Cu-Si-based MG chemistries, *T*_Nose_ is approximately 0.8 times the liquidus temperature (*T*_L_)^5,6,31^. Our simulations reveal a monotonic increase of the cooling rate with the laser scan speed (Fig. 2g inset), which is coupled with the maximum temperature (Fig. 2g). Hence, only scan speeds that result in maximum temperatures exceeding *T*_L_ are considered. The *R*_C_ of Au_55_Cu_25_Si_20_ MG is 3.4 × 10^4^ K s^−1^, as determined from the simulation for a scan speed of 9.2 cm s^−1^.

It should be noted that the simulations do not consider the temperature-dependence of the absorption coefficient of Si, and therefore can be expected to slightly overestimate the cooling rate. These deviations are relatively low for relevant temperatures, as the optical absorption of Si^32^ is four orders of magnitude lower than that of W^33^ at *T*_L_ of Au_55_Cu_25_Si_20_ (654 K)^31^.

To ensure that the measured *R*_C_ reflects the intrinsic behavior of the alloy, one has to consider that at the outline of the heat affected zone resulting from LSA, a solid-liquid interface exists. Here, the unmelted solid could act as a nucleation site during solidification of the melted alloy. Whether or not this situation affects *R*_C_ measurements away from the interface can be concluded by the size of the solidifying grains, which has to be comparable to the melted zone size in order to significantly influence the observed *R*_C_ (Fig. 2b–f). This situation is only present at very slow cooling rates (Fig. 2b), much lower than the intrinsic *R*_C_. Hence, we can conclude that the measured *R*_C_ is independent of the heterogeneous nucleation of from the solid-liquid interface, and is an innate property.

### GFA as a function of composition

**Figure 3:**
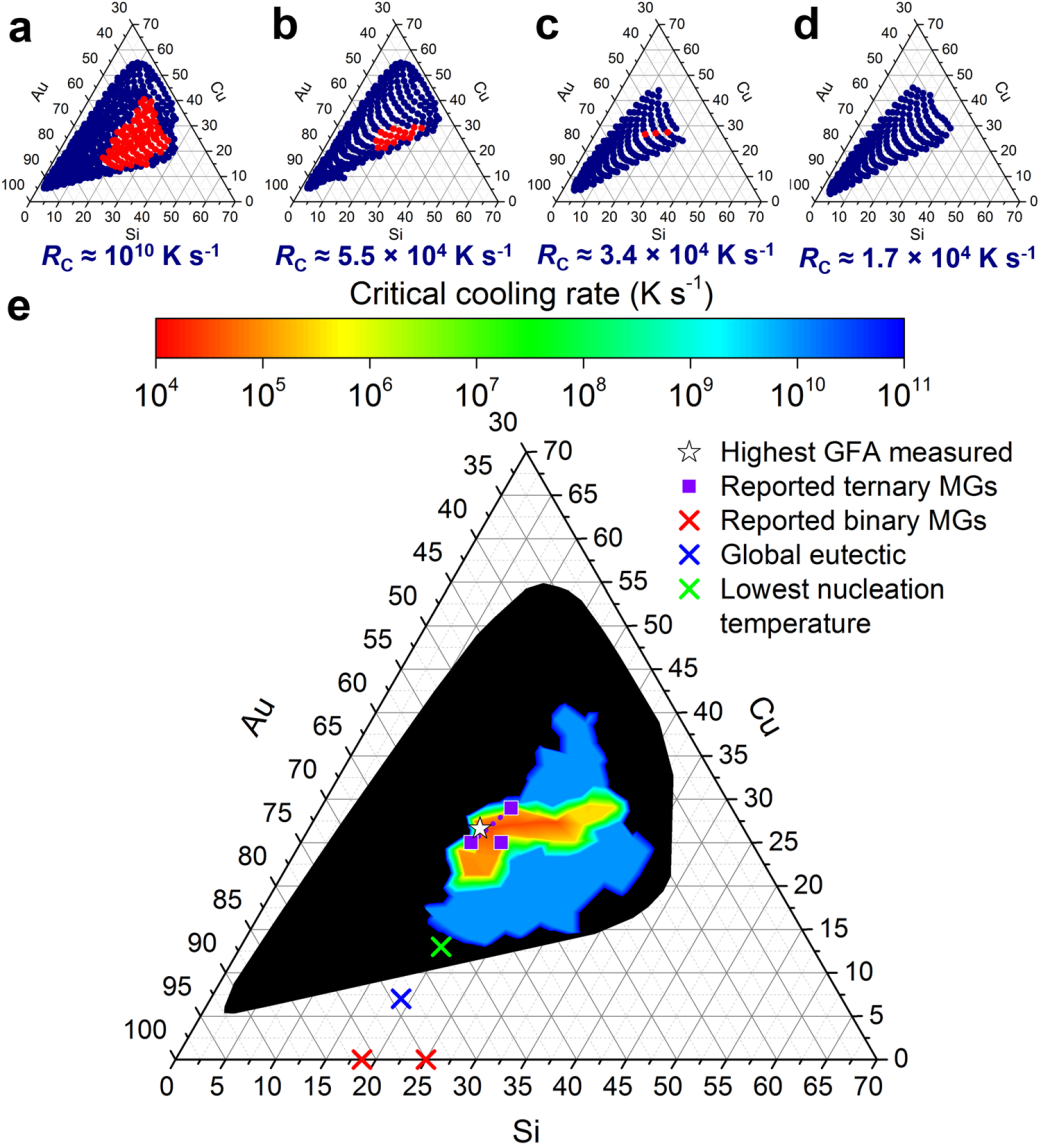
Composition-dependent *R*_C_. Gibbs triangles with red circles depicting the fully amorphous state and navy blue circles depicting states with detectable crystalline phases of (**a**) as-sputtered films, which experienced a cooling rate of 10^10^ K s^−1^, and films that have been laser-treated with scan speeds of (**b**) 11.5 cm s^−1^, (**c**) 9.2 cm s^−1^, and (**d**) 6.9 cm s^−1^. Based on temperature profile simulations (Fig. 2g), the *R*_C_ for the amorphous regions are approximately (**b**) 5.5 × 10^4^ K s^−1^, (**c**) 3.4 × 10^4^ K s^−1^, and (**d**) 1.7 × 10^4^ K s^−1^. (**e**) By compiling the mapped out regions of the amorphous phase as a consequence of multiple laser scan speeds (**a–d**), contours of *R*_C_ as a function of composition can be plotted. The black region represents considered compositions that crystallized, even upon sputtering. The symbols indicate the composition with the highest measured GFA, reported ternary MG compositions with high GFA^21,31^ and dotted lines indicating a range, reported binary MG compositions^36,37^, the global eutectic composition^34^, and the composition with the lowest nucleation temperature^12^. The compositions in all the Gibbs triangles (**a–e**) are in at.%.

The ultimate goal of the developed method is to determine *R*_C_ over large compositional regions. To realize such compositional regions, we fabricate compositional libraries by combinatorial co-sputtering from three sputtering guns in a tetrahedral arrangement^16,20^. Using this method we cover a composition range where Au varies from 32 to 93 at.%, Cu from 5 to 55 at.%, and Si from 1 to 39 at.%. Using EDS and XRD mapping, the composition and structure, respectively, as a function of position on the wafer is determined (Fig. 3). As-sputtered, the entire alloy library experienced a cooling rate of ~10^10^ K s^−1^. This is summarized in the Gibbs triangle indicating amorphous and crystalline regions (Fig. 3a). The as-sputtered state represents the fastest cooling rate in this investigation.

In order to determine *R*_C_ for the alloys with potentially the highest GFA in the Au-Cu-Si system, similar laser scan speeds that were used to determine *R*_C_ of Au_55_Cu_25_Si_20_ (Fig. 2) of 6.9, 9.2, and 11.5 cm s^−1^ were applied for LSA of the alloy libraries (Fig. 3b–d). The cooling rates near *T*_Nose_ that the amorphous regions were exposed to are 1.7 × 10^4^ K s^−1^, 3.4 × 10^4^ K s^−1^, and 5.5 × 10^4^ K s^−1^, respectively. For a scan speed of 11.5 cm s^−1^, the amorphous region shrank significantly in comparison to the one for the as-sputtered data. For example, the maximum of Au variation over which the library remains amorphous reduces from 39 to 66 at.% to 42 to 60 at.%. When the scan speed is further decreased to 9.2 cm s^−1^, the amorphous region narrows further such that only the best glass formers in the compositional space vitrify upon solidification. Here the range in Au content is only 48 to 56 at.%. Vitrified alloys from the 9.2 cm s^−1^ scan speed have *R*_C_ values comparable to Au_55_Cu_25_Si_20_. For a scan speed of 6.9 cm s^−1^, all considered alloys crystallize.

**Figure 4:**
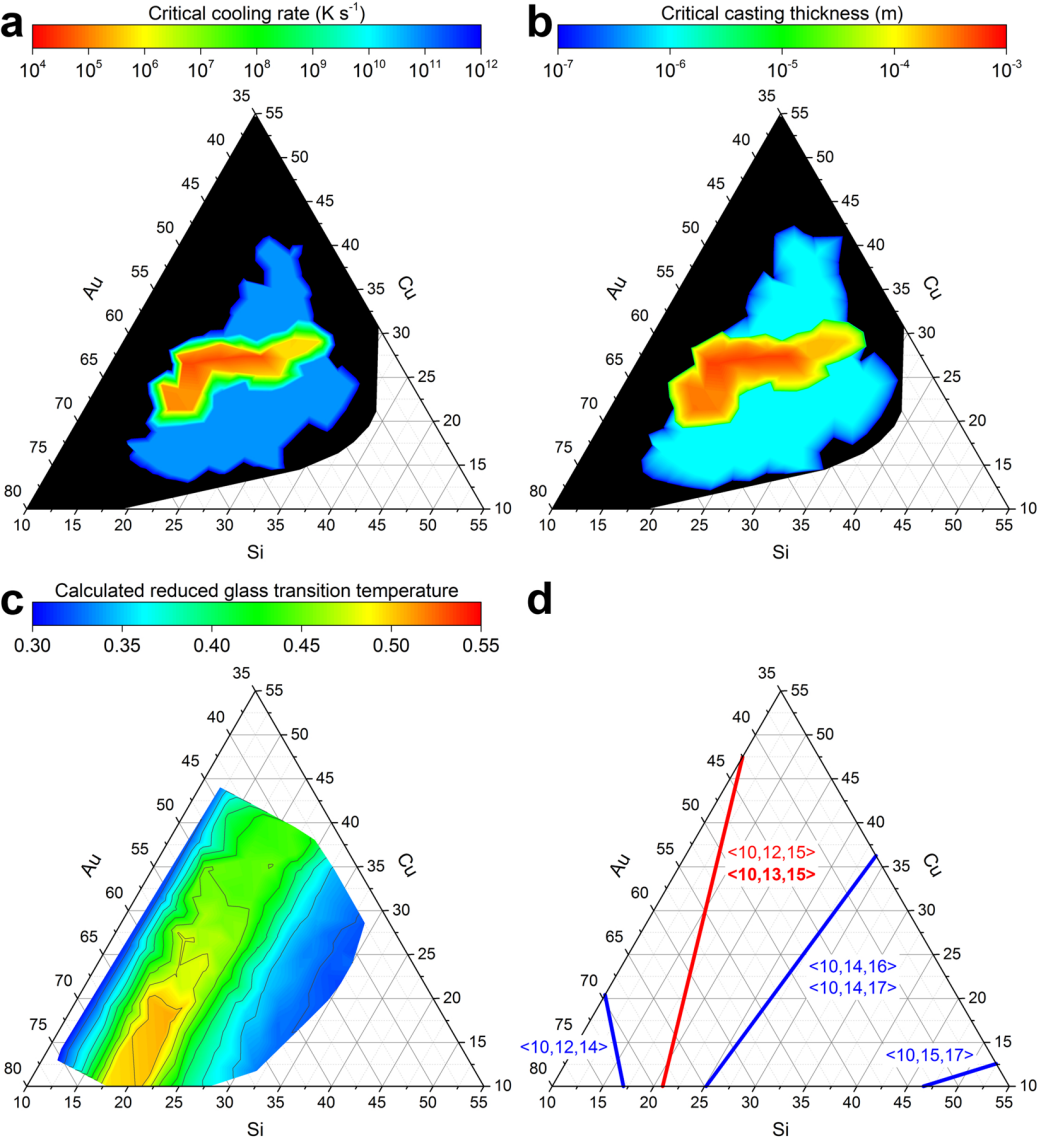
Comparison of composition-dependent GFA obtained from direct measurements with indirect GFA parameters (**a**) Contours of *R*_C_ as a function of composition. (**b**) The measured *R*_C_ is converted to *d*_C_ based on casting into a mold with infinite thermal mass and thermal conductivity, i.e. only the thermal properties of the melt were considered^39^. Given *R*_C_ in K s^−1^, the *d*_C_ in meters is equal to $\sqrt{\frac{0.01}{{R}_{C}}}$. (**c**) *T*_rg_ = *T*_g_/*T*_L_ where *T*_g_ was estimated^41^ by first calculating the elastic moduli^42,43^ and calibrating with a known *T*_g_ that was determined by calorimetry^31^ (calculation details discussed in caption of Supplementary Fig. 1), while *T*_L_ was extracted from the ternary liquidus projection^34^. (**d**) Applying an efficient cluster-packing structural model for metallic glasses^45^, 100% efficiently packed lines for various <*Z*_Si,tot_, *Z*_Cu,tot_, *Z*_Au,tot_> structures were calculated for the composition region of interest. Indicated as a red line is the <10, 13, 15> structure, which is recommended by the authors of the model^45^ for the Au-Cu-Si system.

Across multiple laser scan speeds, *R*_C_ can be calculated from the slowest laser scan speed applied to vitrify the alloy. Once the slowest laser scan speed is determined, the numerical value of *R*_C_ is obtained as the cooling rate at 0.8*T*_L_ from the corresponding simulation (Fig. 2g). *T*_L_ is extracted from the ternary phase diagram^34^. As the final result, *R*_C_ is plotted as a function of composition (Figs 3e and 4a).

## Discussion

The GFA for a large compositional space was directly determined using XRD and EDS mapping of alloy libraries heated with lasers at multiple scan speeds (Figs 3e and 4a). Upon sputtering, the composition range of Au between 39 to 66 at.%, Cu between 13 to 41 at.%, and Si 16 to 35 at.% forms the amorphous phase. The region of the highest GFA (*R*_C_ ≤ 3.4 × 10^4^ K s^−1^) spans from Au_56_Cu_27_Si_17_, along the approximately constant Cu line until Au_48_Cu_28_Si_24_. This region coincides, within margins of error, with Au_55_Cu_25_Si_20_, which is the alloy with the highest previously reported GFA as determined through cumbersome and numerous trial-and-error experiments^31^. The results are also consistent with the region of highest GFA determined by nanocalorimetry, which is the range between Au_58_Cu_25_Si_17_ and Au_52_Cu_29_Si_19_^21^.

From our results, we can draw conclusions about the change of GFA with composition. When the Cu content is maintained between 25 to 30 at.%, *R*_C_ is less sensitive to the Si concentration; *R*_C_ increases gradually from 3.4 × 10^4^ K s^−1^ to about 3 × 10^5^ K s^−1^ when Si content increases from 17 to 29 at.% and then *R*_C_ increases rapidly to over 10^10^ K s^−1^ when the Si concentration is 31 at.% or higher. On the low Si side, GFA drops abruptly once the Si content drops below 16 at.%. For the most extreme case, *R*_C_ changes from 3.4 × 10^4^ K s^−1^ to over 10^10^ K s^−1^ when the Si concentration decreases from 17 to 15 at.%. This implies that within a 2 at.% composition difference, *R*_C_ can change by as much as six orders of magnitude. The degree of variations in GFA with composition are much larger than the variations recently reported for metal-metalloid glass formers^35^. However, it is conceivable that for the proposed method, particularly for this example system, some alloys crystallized rapidly even at ambient or sputtering temperatures (~293 to 323 K). Hence, an alloy that vitrified upon sputtering may have crystallized prior to characterization and would be identified as crystalline upon sputtering. Obviously, this situation is more likely to occur in alloys with glass transition temperatures (*T*_g_) in the vicinity of ambient temperatures such as for binary Au-Si glasses^36^. Evidence for such a scenario is the fact that Au_75_Si_25_^37^ and Au_81.4_Si_18.6_^36^ have both been reported to vitrify upon splat quenching (~10^6^ K s^−1^ ^38^), whereas sputtered (~10^10^ K s^−1^) Au-Si alloys are identified as crystalline. Hence, the composition-dependence of GFA, measured using the proposed method, should be carried out for alloy regions where *T*_g_ is significantly larger than ambient temperature, arguably >340 K (Supplementary Fig. 1).

*R*_C_ can be converted to the critical casting thickness (*d*_C_), the maximum thickness that a material can be made fully amorphous via quenching^39^; an often more practical measure (Fig. 4b). For Au-Cu-Si alloys with the highest GFA (*R*_C_ of 3.4 × 10^4^ K s^−1^), the *d*_C_ is calculated to be 540 μm, consistent with reported values^31^. This confirms the proposed approach and indicates that there is no ternary bulk (*d*_C_ ≥ 1 mm) MG (BMG) in the Au-Cu-Si system.

The availability of *R*_C_ over such a large and unprecedented range of compositions (Figs 3e and 4a) makes it possible to test the validity of indicators of GFA that are widely used and provide insight into motives for glass formation. The reduced glass transition temperature (*T*_rg_ = *T*_g_/*T*_L_), proposed by Turnbull, has been suggested to correlate directly with GFA^40^. However, like for most GFA indicators, *T*_rg_ relies on parameters that are not known *a priori* and therefore have to be estimated (Fig. 4c). Here, *T*_g_ was estimated^41^ as a function of composition by initially calculating the elastic moduli^42,43^ then correlating the elastic moduli values to *T*_g_ which in turn was calibrated with a known *T*_g_ determined by calorimetry^31^; *T*_L_ was obtained from the liquidus projection of the ternary phase diagram^34^. In the composition range under consideration, the estimated *T*_g_ values span from 326 to 376 K (Supplementary Fig. 1), consistent with experimentally determined values^44^. Because the variation in *T*_g_ is significantly less than the variation in *T*_L_, the composition dependence of *T*_rg_ is primarily dictated by the change in *T*_L_.

Based on *T*_rg_, alloys with high GFA are expected to be near eutectic compositions. This is consistent with our reported findings (Figs 3e and 4a). The range of high GFA (*R*_C_ ≤ 10^5^ K s^−1^) corresponds to regions where *T*_rg_ is above 0.4, i.e. between 14 and 27 at.% Si. Moving towards low Si concentrations, at ~15 at.%, the rapid drop in the measured GFA coincides with a steep decline in *T*_rg_. At the higher Si concentrations, the GFA drop is more gradual, despite a substantial decrease in *T*_rg_. Overall, the trends predicted by *T*_rg_ agree broadly with the experimental GFA, although deviations as large as 10 at.% are possible. For example, the composition region of maximum *T*_rg_ does not coincide with the compositions of maximum GFA determined experimentally.

A recently proposed model^45^, based on efficient cluster-packing, has also been tested against the experimentally determined composition dependence of *R*_C_ (Fig. 4d). In short, the model identifies compositions and atomic size differences such that packing clusters are simultaneously efficiently packed about each constituent element. For ternary systems, the most efficiently packed structures form a line in the Gibbs triangle^45^.

The lines of 100% efficiently packed structures were calculated for compositions in the vicinity of the glass-forming range (Fig. 4d)^45,46^. The notation for indicating cluster packing structures is <*Z*_Si,tot_, *Z*_Cu,tot_, *Z*_Au,tot_>, where *Z*_*i*,tot_ is the total number of atoms present in the first shell of a cluster centered about element *i*. The <10, 13, 15> structure has been suggested to represent Au-Cu-Si glass formers^45^. This line coincides with the line for the <10, 12, 15> structure and does indeed intersect with the center of the experimentally determined glass-forming range. Other structures, namely <10, 14, 16> and <10, 14, 17> coincide with the periphery of the experimentally determined glass-forming range. However, the line for the structure predicted by the efficient cluster-packing model, <10, 12, 14>, did not intersect with any glass formers. This comparison suggests that efficient cluster-packing model can assist in reducing the overall composition space, however this reduction is small.

In order to evaluate the effectiveness, practicality, and speed of the proposed technique to experimentally measure GFA over large composition ranges, a comparison with the total number of potential bulk glass forming alloys has to be made. It has been estimated that the number of potential BMG forming alloys is on the order of 10^6^ alloys out of 10^12^ unique alloys with two to five constituent elements^47^. Assuming a fabrication rate of 1000 alloys per day with the proposed method, it would still require over 2,700,000 years to synthesize and characterize the entire alloy space. This comparison suggests that prior to the combinatorial method, a significant reduction in the composition space is required to tackle the complex phenomena of glass formation and to identify many of the yet to be discovered MG and BMG alloys. Here, using theoretical predictions based on parameters that are known *a priori* to identify glass forming systems and reduce the composition space of interest will be powerful^48^. Subsequently these suggested composition ranges would be characterized with our proposed combinatorial method to identify MG and BMG formers. In a final step, a small number of selected alloys, which are suggested by the combinatorial method, would be fabricated in bulk form and characterized by using highly precise and established bulk characterization methods. Thus, the proposed method can be considered the missing link for effective MG discovery.

## Methods

### Sample Fabrication, Laser Heating, and Characterization

The layered film structure is fabricated by confocal DC magnetron co-sputtering (AJA International ATC 2200) with elemental targets with purity exceeding 99.95%. The sputtering guns are arranged in a tetrahedral arrangement at a default angle of 29.8 degrees from the vertical. Prior to any sputtering step, a base pressure level of 10^–6^ Pa and a working pressure of 0.3 Pa of flowing ultra high purity (UHP) Ar is achieved. The substrates were 100 mm diameter, 500 μm thick, double-side polished, intrinsic Si wafers (UniversityWafer, Inc.). Firstly, 30 nm of W was sputtered at 70 W power. Then, 30 nm of Si_3_N_4_ was sputtered by reactive sputtering, using a Si sputtering gun at 70 W under flowing UHP Ar and additionally flowing 10 standard cm^3^ min^−1^ of UHP N_2_ gas. Finally, 1 μm of Au-Cu-Si alloy is co-sputtered with powers of 41 W, 37 W, and 175 W for Au, Cu, and Si, respectively. For a constant composition film, the substrate is rotated during the sputtering process. To fabricate alloy libraries with compositional gradients, the substrates are left stationary. Changing the sputtering power and tilt angles of each specific sputtering gun would alter the compositional spread of the libraries.

The as-sputtered alloy film is first characterized with automated XRD (Rigaku Smartlab) using Cu Kα radiation with a 2 mm beam mask and automated EDS (Oxford Instruments X-Max detector attached to a Zeiss Sigma VP Field Emission scanning electron microscope) to determine the structure and composition, respectively, as a function of position on the wafer. Next, the sample undergoes LSA using a 60 W CO_2_ laser (Universal Laser Systems VLS 6.60) at a set laser scan speed, incident from the heat sink (Si wafer) side. The laser scan lines are spaced 5 mm apart center-to-center to avoid heat affected zone interactions. Finally, the sample is then characterized with XRD and OM (Nikon ME600 with ThorLabs CCD camera).

### Temperature profile simulations

Temperature profile simulations were done using the commercial package COMSOL Multiphysics^®^ for time-dependent thermal finite element method simulations, informed by the experimental results. A 100 mm intrinsic Si wafer was modeled using temperature-dependent thermal conductivity, density, and specific heat capacity^49^. The wafer is assumed to be thermally isotropic. All surfaces are air-cooled with no forced convection (*h* = 5). Ambient temperature and the wafer edge were assumed to be 300 K. The laser was modeled as a Gaussian-shaped surface heat source, where the peak width and power was fitted with experimental measurements of the heat-affected zone size, which was measured using the OM images with ImageJ^50^ software. The thin photo-thermal and alloy layers, having high thermal diffusivities and low thermal masses, are assumed to be isothermal to the top surface of the Si wafer. The temperature profiles for different scan speeds (Fig. 2g) were recorded from one fixed point, located 1.27 cm from the origin of laser incidence and along the path of the laser scan to allow time to reach a stable temperature.

### Data Availability

The datasets generated during and/or analyzed during the current study are available from the corresponding author on reasonable request.

## Electronic supplementary material

Supplementary Information
