# Author Correction: Determination of critical cooling rates in metallic glass forming alloy libraries through laser spike annealing

**DOI:** 10.1038/s41598-018-36256-9

**Published:** 2018-12-12

**Authors:** Punnathat Bordeenithikasem, Jingbei Liu, Sebastian A. Kube, Yanglin Li, Tianxing Ma, B. Ellen Scanley, Christine C. Broadbridge, Joost J. Vlassak, Jonathan P. Singer, Jan Schroers

**Affiliations:** 1 Department of Mechanical Engineering and Materials Science, Yale University, New Haven, Connecticut 06511 USA; 2 Department of Mechanical and Aerospace Engineering, Rutgers University, Piscataway, New Jersey 08854 USA; 3 Department of Physics, Southern Connecticut State University, New Haven, Connecticut 06515 USA; 4 School of Engineering and Applied Science, Harvard University, Cambridge, Massachusetts, 02138 USA

Correction to: *Scientific Reports* 10.1038/s41598-017-07719-2, published online 02 August 2017

The Acknowledgements section in this Article is incomplete.

“This work was funded by National Science Foundation (NSF) DMREF/GOALI 1436268. The authors are grateful for characterization facilities provided by NSF MRSEC DMR 1119826 (CRISP) and the Yale Institute for Nanoscience and Quantum Engineering (YINQE). The authors would like to thank Glenn Weston-Murphy for his help with the laser equipment. The authors would also like to thank Sungwoo Sohn, Frederick J. Walker, Chinedum O. Osuji, Michael Loewenberg, and Corey S. O’Hern for fruitful discussions.”

should read:

“This work was funded by National Science Foundation (NSF) DMREF/GOALI 1436268. The development of the technique was funded through NSF DMR 1609391. The authors would like to thank Glenn Weston-Murphy for his help with the laser equipment. The authors would also like to thank Sungwoo Sohn, Frederick J. Walker, Chinedum O. Osuji, Michael Loewenberg, and Corey S. O’Hern for fruitful discussions.”

