# Supplementary material for: Determination of critical cooling rates in metallic glass forming alloy libraries through laser spike annealing
Source: Sci Rep. 2017 Aug 2;7:7155. doi: 10.1038/s41598-017-07719-2 (PMC5540923; doi:10.1038/s41598-017-07719-2)
Supplement: Supplementary file 1 — Supplementary Information [file 41598_2017_7719_MOESM1_ESM.pdf]

Supplementary Information

Determination of critical cooling rates in metallic glass forming alloy libraries through laser spike annealing

Punnathat Bordeenithikasem[1], Jingbei Liu[1], Sebastian A. Kube[1], Yanglin Li[1], Tianxing Ma[2], B. Ellen Scanley[3], Christine C. Broadbridge[3], Joost J. Vlassak[4], Jonathan P. Singer[2] & Jan Schroers[*,1]

[1]*Department of Mechanical Engineering and Materials Science, Yale University, New Haven, Connecticut 06511, USA*

[2]*Department of Mechanical and Aerospace Engineering, Rutgers University, Piscataway, New Jersey 08854, USA*

[3]*Department of Physics, Southern Connecticut State University, New Haven, Connecticut 06515, USA*

[4]*School of Engineering and Applied Science, Harvard University, Cambridge, Massachusetts 02138, USA*

[*]*Corresponding author:*
Email: jan.schroers@yale.edu
Postal Address: 15 Prospect St., Becton Center 217, New Haven, Connecticut 06511, USA

Supplementary Figures

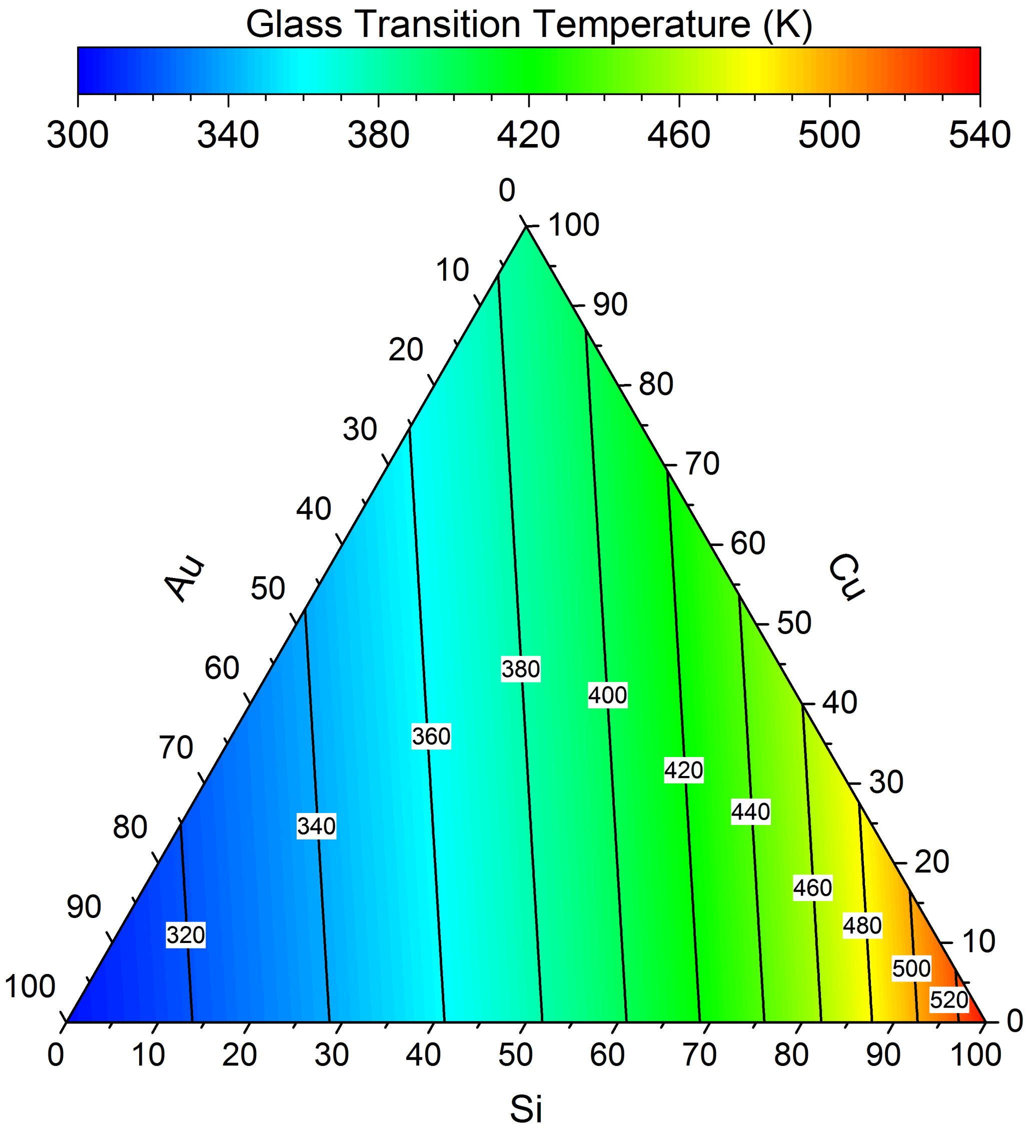

Supplementary Figure 1. Contour plot of the estimated composition-dependent glass transition temperature (T_g) of the Au-Cu-Si system. It has been reported by Wang[1] that in metallic glasses (MGs), $T_g = 2.5E + C$, where E is the elastic modulus and C is an arbitrary constant. The E of MG forming alloys can be calculated using the relation: $E^{-1} = \sum f_i E_i^{-1}$, where f_i is the atomic fraction of constituent element i and E_i is the elastic modulus of corresponding element[2]. For the Au-Cu-Si system, the elastic moduli are taken to be 77 GPa, 110 GPa, and 169 GPa for Au, Cu, and Si

respectively[3]. As a final step, C is determined, by using experimentally determined T_g (e.g. by calorimetry) for an alloy of known composition; i.e. $Au_{55}Cu_{25}Si_{20}$ with a T_g of 348 K[4]. Using the completed formula, T_g as a function of composition is then calculated and plotted.

Supplementary References

1. Wang WH. Elastic moduli and behaviors of metallic glasses. *Journal of Non-Crystalline Solids* **351**, 1481-1485 (2005).

2. Wang WH. Correlations between elastic moduli and properties in bulk metallic glasses. *Journal of Applied Physics* **99**, 093506 (2006).

3. Callister WD, Rethwisch DG. *Materials Science and Engineering: An Introduction*, Ninth edn. Wiley (2014).

4. Schroers J, Lohwongwatana B, Johnson WL, Peker A. Gold based bulk metallic glass. *Applied Physics Letters* **87**, 061912 (2005).
